# The environmental risk factors prior to conception associated with placental abruption: an umbrella review

**DOI:** 10.1186/s13643-022-01915-6

**Published:** 2022-04-01

**Authors:** Ensiyeh Jenabi, Zohreh Salimi, Erfan Ayubi, Saeid Bashirian, Amir Mohammad Salehi

**Affiliations:** 1 Mother and Child Care Research Center, Hamadan University of Medical Sciences, Hamadan, Iran; 2 Autism Spectrum Disorders Research Center, Hamadan University of Medical Sciences, Hamadan, Iran; 3 Social Determinants of Health Research Center, Hamadan University of Medical Sciences, Hamadan, Iran; 4 School of Medicine, Hamadan University of Medical Sciences, Hamadan, Iran

**Keywords:** Placental abruption, Risk factor, Umbrella review

## Abstract

**Background:**

The present umbrella review evaluated risk factors prior to conception associated with placental abruption based on meta-analyses and systematic reviews.

**Methods:**

We searched PubMed, Scopus, and Web of Science until June 25, 2021. All meta-analyses that had focused on assessing the risk factors associated with placental abruption were included. We calculated summary effect estimates, 95% CI, heterogeneity *I*^2^, 95% prediction interval, small-study effects, excess significance biases, and sensitive analysis. The quality of the meta-analyses was evaluated with A Measurement Tool to Assess Systematic Reviews 2 (AMSTAR 2).

**Results:**

There was no risk factor in the present umbrella review with the high level of evidence (class I or II). Eight risk factors including maternal asthma (RR 1.29 95% CI 1.14, 1.47), prior cesarean section (RR 1.38, 95% CI 1.35–1.42), cocaine using (RR 4.55, 95% CI 1.78–6.50), endometriosis (OR 1.40, 95% CI 1.12–1.76), chronic hypertension (OR 3.13, 95% CI 2.04–4.80), advanced maternal age (OR 1.44, 95% CI 1.35–1.54), maternal smoking (OR 1.80, 95% CI 1.75–1.85) (RR 1.65, 95% CI 1.51–1.80), and use of assisted reproductive techniques (ART) (OR 1.87, 95% CI 1.70–2.06) were graded as suggestive evidence (class III). The other four risk factors including pre-pregnancy underweight (OR 1.38, 95% CI 1.12–1.70), preeclampsia (OR 1.73, 95% CI 1.47–2.04), uterine leiomyoma (OR 2.63, 95% CI 1.38–3.88), and marijuana use (OR 1.78, 95% CI 1.32–2.40) were graded as risk factors with weak evidence (class IV).

**Conclusion:**

Maternal asthma, prior cesarean section, cocaine use, endometriosis, chronic hypertension, advanced maternal age, maternal smoking, and use of ART, pre-pregnancy underweight, preeclampsia, uterine leiomyoma, and marijuana use were risk factors associated with placental abruption. Although factors associated with placental abruption have been investigated, the current meta-analytic associations cannot disentangle the complex etiology of placental abruption mainly due to their low quality of evidence.

**Supplementary Information:**

The online version contains supplementary material available at 10.1186/s13643-022-01915-6.

## Background

The complete or partial separation of site implanted placental before delivery is defined as placental abruption [1]. It happens in 0.8 to 1% of births [2]. The etiology of placental abruption remains unclear. Women with placental abruption are at increased risks of perinatal morbidity and mortality, maternal postpartum hemorrhage, shock, and cardiovascular disease [3, 4]. Some meta-analyses reported risk factors associated with placental abruption. Identifying risk factors of placental abruption can help high-risk women who need more careful monitoring during pregnancy. These risk factors include advanced maternal age, cocaine use, marijuana use, maternal smoking, prior cesarean section, endometriosis, assisted reproductive technology (ART) use, low pre-pregnancy body mass index (BMI), hypertensive disorders, uterine leiomyoma, and maternal asthma [5–10].

To our knowledge, no umbrella reviews have been conducted to assess risk factors associated with placental abruption on meta-analyses and systematic reviews. Therefore, the present umbrella review evaluated risk factors prior to conception associated with placental abruption based on meta-analyses and systematic reviews.

## Methods

In this manuscript, we used Preferred Reporting Items for Systematic Reviews and Meta-Analyses (PRISMA) guidelines for conducting the umbrella review and reporting the findings [11]. Also, we used a pre-specified protocol registered at the database of the International Prospective Register of Systematic Reviews with PROSPERO registration number CRD42021265816.

### Inclusion and exclusion criteria

The identified articles through systematic database searching along with the additional articles identified through forward and backward searching were screened for eligible articles, firstly at title-screening level, then at the abstract level, and finally at full-text-level, independently, by EJ and AS. Disagreements were resolved by SB.

We looked for all systematic reviews and meta-analyses that were performed focusing on environmental risk factors prior to conception associated with mothers for placental abruption. The systematic reviews were included if they considered observational studies (cohort, case-control) and included meta-analyses, with no language or date restrictions. Partial or complete placental abruption needs to be clinically diagnosed. Both journal papers and conference full papers were included.

Systematic reviews were excluded if they did not identify environmental risk factor(s) prior to conception for placental abruption. Also, if the information needed to reanalyze the meta-analyses was not included or could not be retrieved, the review was excluded. Animal studies and genetic studies were excluded. Conference abstracts were also excluded. In case there were multiple reviews considering the same risk factor(s), the one that included the largest number of studies was selected. The excluded papers, with reasons to exclude, are listed in supplementary table 2.

### Literature search

PubMed, Scopus, and Web of Science databases were searched from inception to June 25, 2021, with no restrictions on language or date of publication. These databases were searched for systematic reviews and meta-analyses that had focused on risk factors associated with placental abruption. The search strategy and the search terms used for Scopus and PubMed are included in supplementary 1. We identified the following risk factors prior to conception through the systematic search: maternal smoking, advanced maternal age, cocaine usage, marijuana use, prior cesarean section, endometriosis, assisted reproductive technology (ART), pre-pregnancy body mass index (BMI), hypertensive disorders, uterine leiomyoma, and maternal asthma.

### Selection of studies

We used Endnote software to manage the search results. Two authors (EJ and AS) independently searched the databases to identify eligible articles. Then, the reference of the identified articles was manually searched for potential related systematic reviews not identified by the search engines (backward searching). Also, the authors of the identified articles were contacted for their potential works that are missed or not published yet (forward-searching).

### Data extraction and quality assessment

EJ extracted the information and EA checked the extracted data. Disagreement between them was resolved by SB. The included reviews were assessed and the below information was extracted: first author, publication year, risk factor(s), heterogeneity, effect size, sample size, study estimates, *p*-values, study design, participant demographics, baseline characteristics, and finally, metrics used in their own included articles (odds ratio, related risk, hazard ratio), and all the necessary information needed for re-analysis such as the contingency table. If this information was not included in the meta-analysis, the original articles used in that meta-analysis were retrieved or their authors were contacted. Data storage was on Microsoft Excel spreadsheets.

EJ and AS independently determined the quality of the identified papers using AMSTAR2 [12]. Disagreement between the assessors was resolved by SB. In AMSTAR2, 16 questions are involved, namely (1) PICO (P: patient, I: intervention, C: comparison, O: outcomes) considered in the research question and inclusion criteria? (2) Protocol was established beforehand? Any deviations? (3) Explained if/why only certain study designs were included? (4) Comprehensive search? (5) Two persons performed the search? (6) Two persons extracted the data? Provided the exclusion list with reasons? (7) All details of the included papers presented? (8) Proper technique for assessing the risk of bias? (9) Reported the sources of funding? (10) Appropriate statistical methods? (11) Assessment of the potential impact of risk of bias in individual studies on the results of the meta-analysis? (12) Assessment of the potential impact of risk of bias in individual studies on the discussion of the meta-analysis? (13) Discussion of heterogeneity of the results of meta-analysis? (14) Investigation of publication bias? (15) Have they influenced the results? (16) Potential conflict of interest reported?

Each of the above items was scored as yes, partial yes, or no, and items 2, 4, 7, 9, 11, 13, and 15 are rated as critical, with a higher weight in scoring. The overall score was used to rate the quality of the meta-analyses as high, moderate, low, or critically low.

### Data analysis

ZS performed the statistical analyses using R Version 4.0.5. R packages that were used included Metafor, xlsx, epiR, ConfoundedMeta, and reporter. All statistical tests were two-tailed. The included meta-analyses were re-analyzed by extracting the metrics such as contingency table, *p*-value, and sample size from the original papers. Where this information was not provided, the authors of the original papers were contacted for this information. If the information needed could not be retrieved, the original papers were excluded from the re-analyses.

We used the random-effect model to re-analyze the included meta-analyses and found odds ratio, relative risk, or hazard ratio, based on the effect estimate used for each meta-analysis, and summary effect size, 95% confidence interval, and *p*-values were found. Statistical significance was ascertained at *p* < 0.05. We used Cochrane’s *Q* test to calculate *I*^2^ which is a measure that determines the heterogeneity between studies so that a high heterogeneity is indicated by *I*^2^ > 50%. Also, we found the 95% prediction interval, which is the interval that future mean effect estimates will lie in 95% of the time. Additionally, the Egger regression asymmetry test was used to determine if there exists a small study effect in the original studies. The small study effect deals with the phenomenon that smaller studies typically report larger effect sizes. Furthermore, an excess significance bias test was applied for statistically significant original studies to see if their reported significant results are more than their expected number of significant results, based on the power of their studies (*p* < 0.05).

### Sensitivity analysis

Sensitivity analysis was done for all meta-analyses using Mathur’s method [13]. This method was chosen over using credibility ceilings because the correctness of the latter method was questioned by Mathur et al. [14]. We run this analysis to look for potential spurious significance in the reported results that have been caused by any potential biases or confounders that have been missed when running the experiments. Using this method, we obtain a bias factor, and a confounding association strength paired with it, for each meta-analysis that would be able to reduce the percentage of studies with acceptable effect size, set by us, to less than a tolerable threshold, also set by us. This will help us judge the robustness of the meta-analyses to unmeasured confounders and biases.

### Grading the evidence

Similar to the previously-published umbrella reviews [15, 16], the evidence presented in each meta-analysis here was classified as convincing, highly suggestive, suggestive, weak, and not significant, as below:

Convincing (class I): *p*-value of the random-effect model< 10^−6^, # cases > 1000, no sign of excess significant bias or small study effect, prediction interval not including the null value, robustness to unmeasured confounding, significant result (*p* < 0.05) for the largest study, and *I*^2^ < 50%

Highly suggestive (class II): *p*-value of the random-effect model< 10^−6^, # cases > 1000 (or more than 20,000 participants for continuous outcomes), and significant result (*p* < 0.05) for the largest study

Suggestive (class III): *p*-value of the random-effect model < 10^**−**3^ and # cases > 1000

Weak (class IV): evidence was assigned to the remaining significant association with a *p*-value of the random-effect model < 0.05

(Class V): *p*-value of the random-effect model > 0.05

## Results

**Fig. 1:**
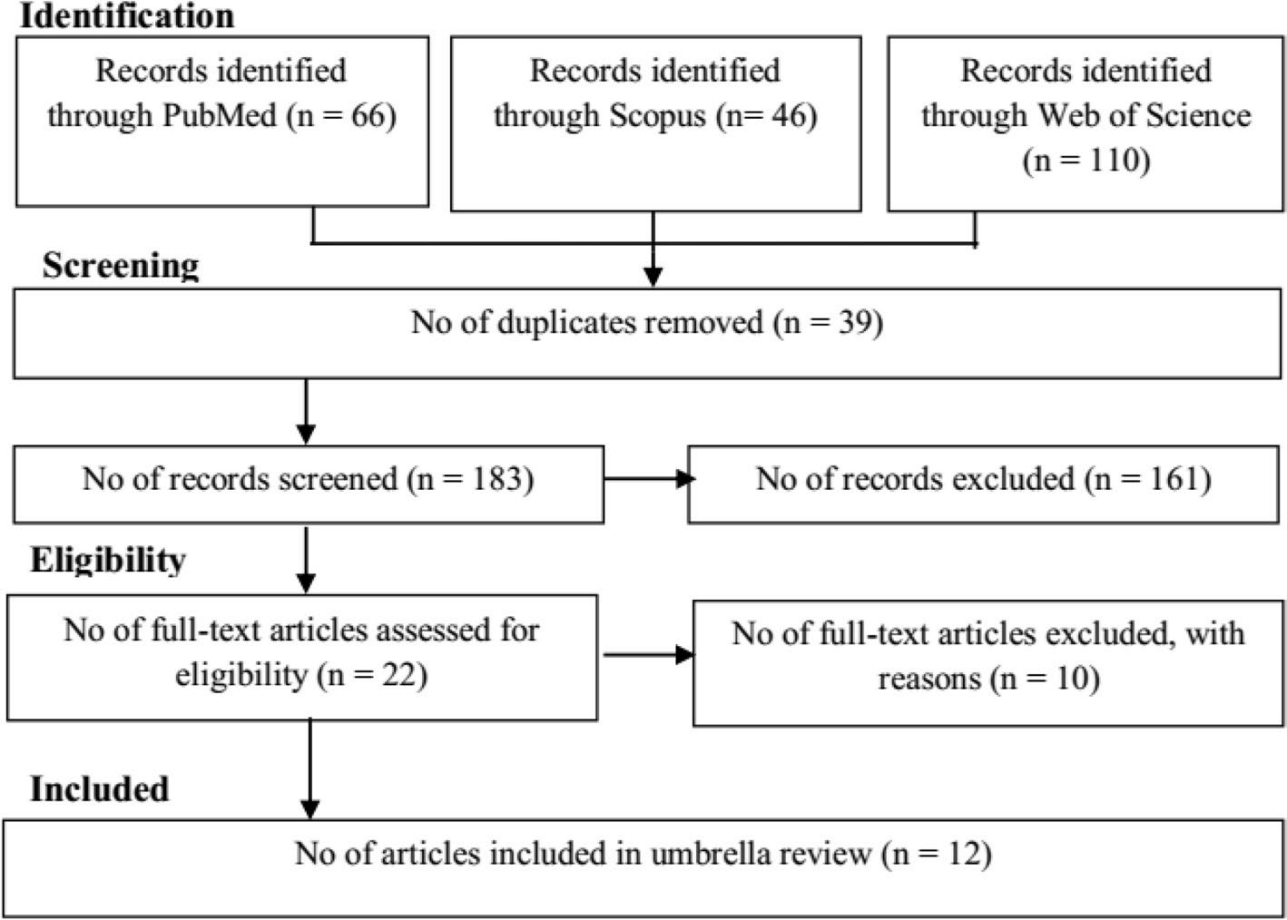
Flowchart of the included meta-analyses in umbrella review

In the present umbrella review, 572 studies until June 25, 2021, were identified. In total, 12 studies that were eligible for inclusion in the present umbrella review (Fig. 1) were included. These 12 eligible studies [4–10, 17–21] provided 15 meta-analyses (Table 1) with 419,460 placental abruption cases and 40,695,813 population. The studies that were included in meta-analyses had cohort or case-control designs. In the present umbrella review, 115 cohort studies and 35 studies based on case-control were included.

**Table 1:** Risk factors for included meta-analysis in the present umbrella review

Risk factors	Source (year)	Number of population	Number of included studies	Study design	Effect metrics	Random effect summary estimate	Credibility of evidence	AMSTAR2 quality
Maternal asthma	Wang, 2014	1,377,063	11	Cohort	Relative risk	1.29 (1.14, 1.47)	Suggestive	Critically low
Maternal pre-pregnancy BMI Underweight Overweight Obese	Adane, 2019	114,792	9	Cohort/case-control	Odds ratio	1.38 (1.12, 1.70) 0.84 (0.76, 0.93) 0.77 (0.68, 0.87)	Weak Weak Suggestive	Critically low
Prior cesarean section	Klar, 2014	5,454,621	8	Cohort/case-control	Relative risk	1.38 (1.35, 1.42)	Suggestive	Critically low
Cocaine use	Addis, 2001	21,881	13	Cohort/case-control	Relative risk	4.55 (3.19, 6.50)	Suggestive	Low
Endometriosis	Breintoft, 2021	7,320,658	8	Cohort	Odds ratio	1.40 (1.12, 1.76)	Suggestive	Critically low
Fetal sex	Broere-Brown, 2020	3,130,530	13	Cohort/case-control	Odds ratio	1.07 (0.93, 1.23)	NS	Critically low
Chronic hypertension	Ananth, 1996	517,382	8	Cohort/case-control	Odds ratio	3.13 (2.04, 4.80)	Suggestive	Critically low
preeclampsia	Ananth,1996	660,979	12	Cohort/case-control	Odds ratio	1.73 (1.47, 2.04)	Weak	Critically low
Uterine leiomyoma	Jenabi, 2017	232,024	9	Cohort/case-control	Odds ratio	2.63 (1.38, 3.88)	Weak	Critically low
Marijuana use	Conner, 2016	22,863	5	Cohort/case-control	Odds ratio	1.78 (1.32, 2.40)	Weak	Critically low
Advanced maternal age	Martinelli, 2018	20,684,077	14	Cohort	Odds ratio	1.44 (1.35, 1.54)	Suggestive	Critically low
Maternal smoking	Shobeiri, 2017	4,309,610	27	Cohort/case-control	Odds ratio Relative risk	1.80 (1.75, 1.85) 1.65 (1.51, 1.80)	Suggestive	Critically low
Use of ART	Vermey, 2019	1,158,943	14	Cohort	Odds ratio	1.87 (1.70, 2.06)	Suggestive	Critically low

*ART* assisted reproductive techniques

We identified 15 factors in the included meta-analyses: maternal asthma, pre-pregnancy underweight, pre-pregnancy overweight, pre-pregnancy obese, prior cesarean section, cocaine use, endometriosis, fetal sex, chronic hypertension, preeclampsia, uterine leiomyoma, marijuana use, advanced maternal age, maternal smoking, and use of assisted reproductive techniques (ART).

Out of the 15 associations in the present umbrella review, 12 associations were statistically significant using the random-effects model (*p* < 0.001), 12 studies included at least 1000 placental abruption cases, eight studies reported heterogeneity (*I*^2^) less than 50%, two studies had small study effects, and six had excess significance bias (Table 2).

**Table 2:** The credibility of the evidence in meta-analyses included studies

Risk factors	Number of cases	Summary associations (*p*-value) per random-effects calculations	Small-study effects (*p*-value for Egger)	Excess of significance bias (*p*-value)	Prediction intervals	Largest study nominally significant (*p*-value)	Heterogeneity (*I*^2^%)	Sensitivity analysis	Classification
Maternal asthma	1451	0.0002	0.931	0.102	1.05–1.62	0.667	44.8	*T* = 1.212, *G* = 1.719	Suggestive
Maternal pre-pregnancy BMI Underweight Overweight Obese	773 666 1036	0.0036 0.0007 0.0002	0.844 0.470 0.599	0.252 0.002 0.277	0.51–16.03 0.75–0.93 0.81–0.94	< 0.01 0.007 < 0.01	55.2 0.0 28.2	*T* = 1.389, *G* = 2.124 *T* = 1.389, *G* = 2.124 T = 1.333, *G* = 2.001	Weak Weak Suggestive
Prior cesarean section	29,001	0.0003	0.140	0.002	0.85–6.50	< 0.0001	0.0	*T* = 2.160, *G* = 3.743	Suggestive
Cocaine use	1123	< 0.0001	0.953	0.003	2.39–5.68	0.001	0.0	*T* = 3.199, *G* = 5.853	Suggestive
Endometriosis	33,378	0.0001	9.929	0.238	0.94–2.50	< 0.0001	82	*T* = 1.454, *G* = 2.266	Suggestive
Fetal sex	21,396	0.7710	0.544	0.741	0.65–1.62	< 0.001	92.9	*T* = 0.995, *G* = Nan	NS
Chronic hypertension	1539	< 0.0001	0.046	0.042	1.67–3.40	0.0006	0.0	*T* = 2.414, *G* = 4.262	Suggestive
preeclampsia	5622	0.0338	0	0.901	1.04–2.50	0.04	0.0	*T* = 1.379, *G* = 2.102	Weak
Uterine leiomyoma	5137	0.0036	0.915	0.602	0.51–16.03	< 0.01	82.6	*T* = 2.182, *G* = 3.788	Weak
Marijuana use	488	0.0002	0.940	0.003	1.30– 2.37	0.026	0.0	*T* = 1.614, *G* = 2.609	Weak
Advanced maternal age	215,829	< 0.0001	0.035	0.291	1.12–1.51	< 0.0001	88.3	*T* = 1.608, *G* = 2.598	Suggestive
Maternal smoking	26,038	< 0.0001	0.477	0.001	1.49–2.32	< 0.01	65.8	*T* = 1.790, *G* = 2.980	Suggestive
Use of ART	75,983	0.0009	0.761	0.772	0.70–3.88	< 0.0001	36	*T* = 1.207, *G* = 1.708	Suggestive

*BMI* body mass index, *ART* assisted reproductive techniques

In sensitivity analyses, the results of 10 meta-analyses were relatively sensitive to unmeasured confounding, considering a bias factor of less than 1.75 in each of their included studies, which was needed to reduce the percentage of studies with a true odds ratio of greater than 1.1 to less than 20%. These factors that are sensitive to unmeasured confounding were maternal asthma, pre-pregnancy underweight, pre-pregnancy overweight, pre-pregnancy obese, endometriosis, fetal sex, preeclampsia, marijuana use, advanced maternal age, and use of ART. However, only three factors (pre-pregnancy overweight, pre-pregnancy obese, and fetal sex) were not statistically confirmed as a risk factor.

The other four factors (prior cesarean section, cocaine using, uterine leiomyoma, maternal smoking) were relatively robust to unmeasured confounding, considering a bias factor of more than 1.9 for each of their included studies was needed to reduce the proportion of studies with a true odds ratio greater than 1.1 to less than 10% (20% in the case of smaller meta-analyses) (Table 2).

In the present umbrella review, there was no risk factor with the high level of evidence (class I or II). Eight risk factors including maternal asthma (RR 1.29 95% CI 1.14, 1.47), prior cesarean section (RR 1.38, 95% CI 1.35–1.42), cocaine using (RR 4.55, 95% CI 1.78–6.50), endometriosis (OR 1.40, 95% CI 1.12–1.76), chronic hypertension (OR 3.13, 95% CI 2.04–4.80), advanced maternal age (OR 1.44, 95% CI 1.35–1.54), maternal smoking (OR 1.80, 95% CI 1.75–1.85) (RR 1.65, 95% CI 1.51–1.80), and use of ART (OR 1.87, 95% CI 1.70–2.06) were graded as suggestive evidence (class III).

**Fig. 2:**
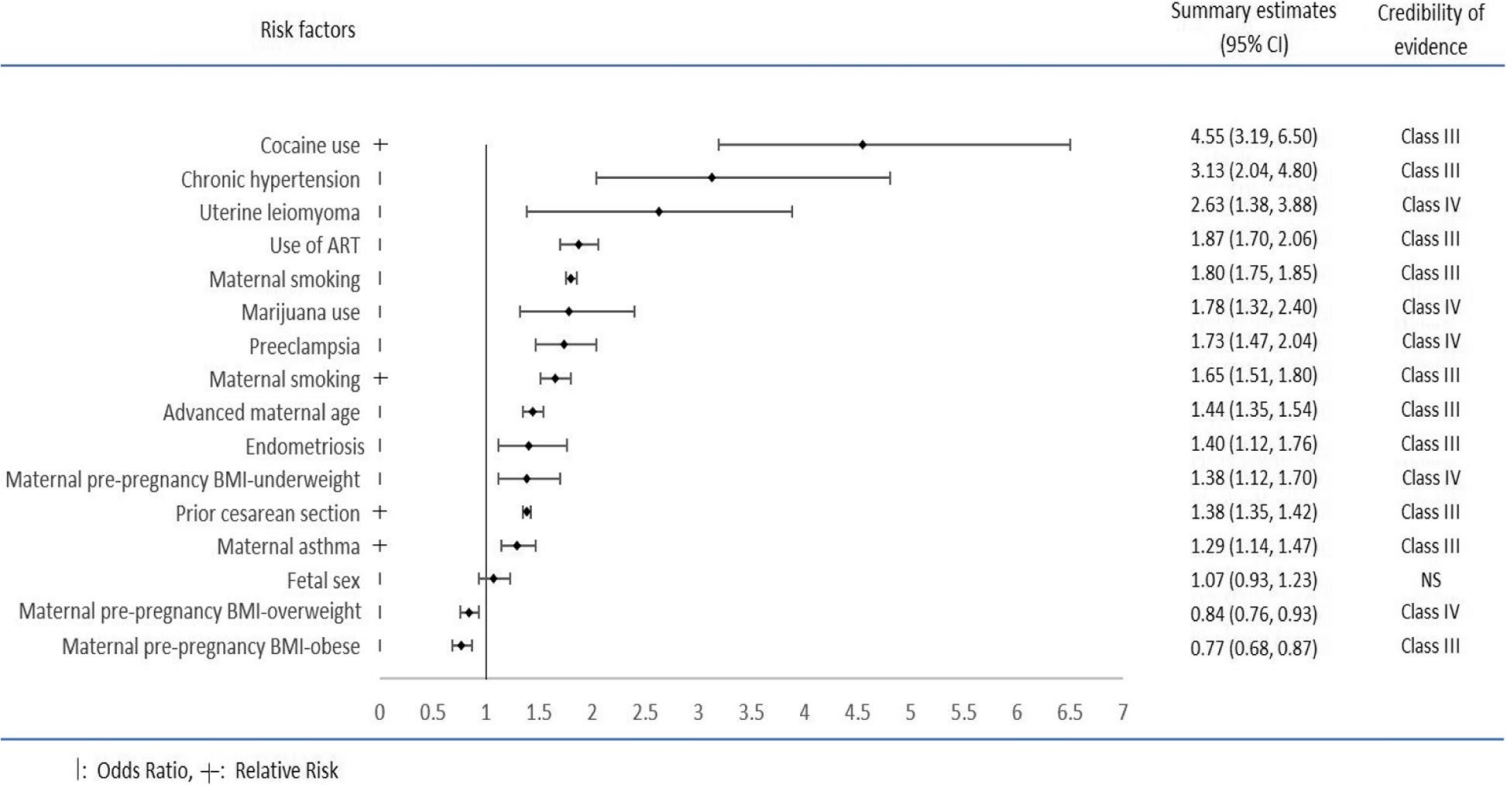
Summary estimates of meta-analyses of potential environmental risk factors for placental abruption

The other four risk factors including pre-pregnancy underweight (OR 1.38, 95% CI 1.12–1.70), preeclampsia (OR 1.73, 95% CI 1.47–2.04), uterine leiomyoma (OR 2.63, 95% CI 1.38–3.88), and Marijuana use (OR 1.78, 95% CI 1.32–2.40) were graded as risk factors with weak evidence (class IV). Fetal sex (OR 1.07, 95% CI 0.93–1.23) was not confirmed as a risk factor for placental abruption (not significant). Pre-pregnancy overweight (OR 0.84, 95% CI 0.76–0.93) and obese (OR 0.77, 95% CI 0.68–0.87) were protective factors for placental abruption graded in class IV and class III, respectively (Table 1, Fig. 2).

The quality of all meta-analyses except for a meta-analysis, based on AMSTAR 2, was critically low (Table 1 and S3).

## Discussion

We identified 15 meta-analyses of cohort or case-control studies, including 419,460 placental abruption cases and 40,695,813 population. The results of this umbrella review study provide that a constellation of 15 risk factors exist for placental abruption as follow; maternal asthma, prior cesarean section, cocaine use, endometriosis, chronic hypertension, obesity, advanced maternal age, and use of ART with suggestive (class III) credibility and underweight, overweight, preeclampsia, uterine leiomyoma, and marijuana use with weak (class IV) credibility.

There were no meta-analyses with a high level of credibility, including convincing (class I) and highly suggestive (class II). It means that further large cohort studies with homogeneous study groups and with a consistent and reliable definition of risk factors and placental abruption are needed. Moreover, studies included in each meta-analysis on risk factors of placental abruption should involve both studies with statistically significant and non-significant results for precluding potential excess significance bias [22].

The mechanisms involved in the association of placental abruption and risk factors are not well understood. Previous studies suggest that subfertility plays a role in abnormal placentation in singleton pregnancies. Therefore, the use of ART pregnancy can be associated with placental abruption [23]. Smoking due to hypoxemia-induced changes in the uteroplacental circulation may play a role in placental abruption through the vasoconstrictive effects of nicotine on the uterine and umbilical arteries [24]. Underweight was one of the risk factors for placental abruption. Underweight women are more prone to micronutrient deficiencies and therefore have fewer reserves to meet the additional nutritional needs of pregnancy [25]. These adverse conditions may contribute to poor placental growth and be led to the risk of placental abruption [26]. Endometriosis with the thickening of the junctional zone can lead to abnormal regeneration of the spiral arteries and defective deep placentation, especially in women with advanced stages of endometriosis [27]. The increased risk of placental abruption in women with uterine leiomyoma appears to be due to interference or distortion of the normal perfusion of the placental site [28].

Association between maternal smoking during pregnancy and placental abruption was studied more than other risk factors so that in the study by Shobeiri et al. [10], 27 studies were included in the meta-analysis. The pooled OR and RR for the effect of maternal smoking on placental abruption were 1.80 and 1.65, respectively. The difference between OR and RR is negligible when the outcome of interest is rare in all levels of exposure of interest [29], so it seems that the frequency of placental abruption is low among both mothers who smoked and not smoked and OR will approximate the RR.

This study found a fourfold increased risk of placental abruption among those who use cocaine versus those who do not use it. According to AMSTAR, overall confidence in the results was low, and it can be inferred that the degree of selection bias, information bias, and/or confounding did not control or adjust among individual studies that address the association between cocaine use and placental abruption. In other words, the cocaine can be measured using different methods, e.g., urine analysis, maternal interview, chart review, or it may be cocaine alone and polydrug including cocaine [30]. The different methods of ascertainment of cocaine use induce a degree of information bias in the results. The sensitivity analysis results emphasize the degree of different bias in cocaine use and placental abruption pathway (*T* = 3.19 and *G* = 5.85). Because marijuana was positively associated with placental abruption (pooled OR = 1.78), it seems combinations of cocaine and marijuana have a synergistic effect on placental abruption. It is not an unexpected fact that a woman during pregnancy experiences multiple substances such as illicit drugs, cocaine, and marijuana [31]. One of the essential subjects that should be considered when interpreting the effect of smoking, cocaine, and marijuana use on each adverse outcome is attention to dose-response associations. In other words, the effect estimates for recreational and regular users may be different. The need for attention to the dose-response association is introduced as an essential gap when interpreting associations, e.g., maternal smoking and later adverse outcomes [32]. Further large individual studies are needed that smoking, cocaine, and marijuana use during pregnancy were measured in continuous form and not categorical level and in following, dose-response meta-analyses to be done on the observed data.

After cocaine use, chronic hypertension had the strongest effect on placental abruption (pooled OR = 3.13). In the meta-analysis by Ananth et al. [20], women with chronic hypertension had a 3-fold increase risk of placental abruption. Moreover, they found the results can be modified by country, study design, source of information, and incidence of placental abruption [20]. For example, they mentioned that the number of placental abruption was two times that in case-control studies and higher estimate OR from case-control studies (3.88) in comparison to effect estimate from cohort studies may be the result of reporting bias [20].

Although a dose-response pattern was found between maternal pre-pregnancy BMI and placental abruption, underweight as a risk factor and overweight and obesity as protective factors, respectively [4]; however, a correct decision about the association between pre-pregnancy BMI and placental abruption requires considering confounders in the causal pathway of the two aforementioned variables. For example, previous studies have suggested variables including micronutrient deficiencies [33], lifestyle, underlying medical conditions, and smoking during pregnancy [34–36] can be considered as confounders in the pathway. Nearly 70% of individual studies included in the meta-analysis by Adane et al. [33] that address the association between pre-pregnancy BMI and placental abruption did not adequately address the confounder’s adjustment.

Although this study was the first umbrella review to summarize evidence about the etiology of placental abruption, several issues should be considered when interpreting the results. A valid inference about the association can be obtained by considering more databases and grey literature. Definition and ascertainment of the variables of interest should be clearly and homogenous across all individual studies. Stratification of evidence may be along with the degree of error because, for example, risk factors, e.g., uterine leiomyoma, may have a notable effect on placental abruption (OR = 2.63). However, it may be evaluated in a few studies (9 studies), leading to stratified as class IV evidence.

## Conclusion

Eight risk factors including maternal asthma, prior cesarean section, cocaine use, endometriosis, chronic hypertension, advanced maternal age, maternal smoking, and use of ART were graded as suggestive evidence (class III). The other four risk factors including pre-pregnancy underweight, preeclampsia, uterine leiomyoma, and marijuana use were graded as risk factors with weak evidence (class IV). Pre-pregnancy overweight and obesity were protective factors for placental abruption. Although factors associated with placental abruption have been investigated, the current meta-analytic associations cannot disentangle the complex etiology of placental abruption mainly due to their low quality of evidence.

## Supplementary Information

**Additional file 1: S1.** Search strategy. **S 2.** Excluded references with reasons. **S 3.** Quality of studies based on AMSTAR2 items.

## Data Availability

Not applicable.

## Abbreviations

AMSTAR 2: A Measurement Tool to Assess Systematic Reviews
ART: Assisted reproductive technology
PRISMA: Preferred Reporting Items for Systematic Reviews and Meta-Analyses
BMI: Body mass index

## References

[1] Tikkanen M (2011). Placental abruption: epidemiology, risk factors and consequences. Acta Obstet Gynecol Scand.

[2] Arnold DL, Williams MA, Miller RS, Qiu C, Sorensen TK (2009). Iron deficiency anemia, cigarette smoking and risk of abruptio placentale. J Obstet Gynaecol Res.

[3] Downes KL, Shenassa ED, Grantz KL (2017). Neonatal outcomes associated with placental abruption. Am J Epidemiol.

[4] Adane AA, Shepherd CC, Lim FJ, White SW, Farrant BM, Bailey HD (2019). The impact of pre-pregnancy body mass index and gestational weight gain on placental abruption risk: a systematic review and meta-analysis. Arch Gynecol Obstet.

[5] Klar M, Michels KB (2014). Cesarean section and placental disorders in subsequent pregnancies - a meta-analysis. J Perinat Med.

[6] Jenabi E, Ebrahimzadeh ZS (2017). The association between uterine leiomyoma and placental abruption: A meta-analysis. J Maternal-Fetal Neonatal Med.

[7] Martinelli KG, Garcia EM, Neto ETD, da Gama SGN. Advanced maternal age and its association with placental praevia and placental abruption: a meta-analysis. Cadernos De Saude Publica. 2018;34(2). 10.1590/0102-311X00206116 29489954

[8] Vermey BG, Buchanan A, Chambers GM, Kolibianakis EM, Bosdou J, Chapman MG (2019). Are singleton pregnancies after assisted reproduction technology (ART) associated with a higher risk of placental anomalies compared with non-ART singleton pregnancies? A systematic review and meta-analysis. Bjog-an Int J Obstet Gynaecol.

[9] Wang G, Murphy VE, Namazy J, Powell H, Schatz M, Chambers C (2014). The risk of maternal and placental complications in pregnant women with asthma: a systematic review and meta-analysis. J Maternal-Fetal Neonatal Med.

[10] Shobeiri F, Masoumi SZ, Jenabi E (2017). The association between maternal smoking and placental abruption: a meta-analysis. J Maternal-Fetal Neonatal Med.

[11] Moher D, Shamseer L, Clarke M, Ghersi D, Liberati A, Petticrew M (2015). Preferred reporting items for systematic review and meta-analysis protocols (PRISMA-P) 2015 statement. Syst Rev.

[12] Shea BJ, Reeves BC, Wells G, Thuku M, Hamel C, Moran J, et al. AMSTAR 2: a critical appraisal tool for systematic reviews that include randomised or non-randomised studies of healthcare interventions, or both. BMJ. 2017;358. 10.1136/bmj.j4008 PMC5833365 28935701

[13] Mathur MB, VanderWeele TJ (2020). Sensitivity analysis for unmeasured confounding in meta-analyses. J Am Stat Assoc.

[14] Mathur MB, VanderWeele TJ (2020). Controversy and debate on credibility ceilings. Paper 1: Fundamental problems with the “credibility ceiling” method for meta-analyses. J Clin Epidemiol.

[15] Kim JY, Son MJ, Son CY, Radua J, Eisenhut M, Gressier F (2019). Environmental risk factors and biomarkers for autism spectrum disorder: an umbrella review of the evidence. Lancet Psychiatry.

[16] Jenabi E, Salimi Z, Bashirian S, Khazaei S, Ayubi E. The risk factors associated with placental previa: an umbrella review. Placental. 2021. 10.1016/j.placenta.2021.10.009 34768164

[17] Addis A, Moretti ME, Ahmed Syed F, Einarson TR, Koren G. Fetal effects of cocaine: an updated meta-analysis. Reprod Toxicol. 2001;15(4):341-69. 10.1016/s0890-6238(01)00136-8 11489591

[18] Breintoft K, Pinnerup R, Henriksen TB, Rytter D, Uldbjerg N, Forman A (2021). Endometriosis and risk of adverse pregnancy outcome: a systematic review and meta-analysis. J Clin Med.

[19] Broere-Brown ZA, Adank MC, Benschop L, Tielemans M, Muka T, Gonçalves R, et al. Fetal sex and maternal pregnancy outcomes: a systematic review and meta-analysis. Biol Sex Differ 2020;11(1):1-20. 10.1186/s13293-020-00299-3 PMC7216628 32393396

[20] Ananth CV, Savitz DA, Williams MA (1996). Placental abruption and its association with hypertension and prolonged rupture of membranes: a methodologic review and meta-analysis. Obstet Gynecol.

[21] Conner SN, Bedell V, Lipsey K, Macones GA, Cahill AG, Tuuli MG (2016). Maternal marijuana use and adverse neonatal outcomes. Obstet Gynecol.

[22] Fusar-Poli P, Radua J (2018). Ten simple rules for conducting umbrella reviews. Evid Based Ment Health.

[23] Luke B, Gopal D, Cabral H, Stern JE, Diop H. Pregnancy, birth, and infant outcomes by maternal fertility status: the Massachusetts Outcomes Study of Assisted Reproductive Technology. Am J obstet Gynecol. 2017;217(3):327. e1-. e14. 10.1016/j.ajog.2017.04.006 PMC5581226 28400311

[24] Tikkanen M, Surcel HM, Bloigu A, Nuutila M, Ylikorkala O, Hiilesmaa V (2010). Self-reported smoking habits and serum cotinine levels in women with placental abruption. Acta Obstet Gynecol Scand.

[25] Truong YN, Yee LM, Caughey AB, Cheng YW. Weight gain in pregnancy: does the Institute of Medicine have it right? Am J Obstet Gynecol. 2015;212(3):362. e1-. e8. 10.1016/j.ajog.2015.01.027 25725659

[26] Deutsch AB, Alio AP, Salihu HM, Spellacy WN (2010). Increased risk of placental abruption in underweight women. Am J Perinatol.

[27] Landi S, Mereu L, Pontrelli G, Stepniewska A, Romano L, Tateo S (2008). The influence of adenomyosis in patients laparoscopically treated for deep endometriosis. J Minim Invasive Gynecol.

[28] Rice JP, Kay HH, Mahony BS (1989). The clinical significance of uterine leiomyomas in pregnancy. Am J Obstet Gynecol.

[29] Cummings P (2009). The relative merits of risk ratios and odds ratios. Arch Pediatr Adolesc Med.

[30] Addis A, Moretti ME, Syed FA, Einarson TR, Koren G (2001). Fetal effects of cocaine: an updated meta-analysis. Reprod Toxicol.

[31] Ebrahim SH, Gfroerer J (2003). Pregnancy-related substance use in the United States during 1996–1998. Obstet Gynecol.

[32] Avşar TS, McLeod H, Jackson L (2021). Health outcomes of smoking during pregnancy and the postpartum period: an umbrella review. BMC Pregnancy Childbirth.

[33] Torheim LE, Ferguson EL, Penrose K, Arimond M (2010). Women in resource-poor settings are at risk of inadequate intakes of multiple micronutrients. J Nutr.

[34] Audrain-McGovern J, Benowitz N (2011). Cigarette smoking, nicotine, and body weight. Clin Pharmacol Therapeutics.

[35] Ding X-X, Xu S-J, Hao J-H, Huang K, Su P-Y, Tao F-B (2016). Maternal pre-pregnancy BMI and adverse pregnancy outcomes among Chinese women: results from the C-ABCS. J Obstet Gynaecol.

[36] Ra S-P, Spence D, Cardwell C, Hunter A, Holmes V (2013). The impact of body mass index on maternal and neonatal outcomes: a retrospective study in a UK obstetric population, 2004–2011. BJOG.

