# Supplementary material for: The environmental risk factors prior to conception associated with placental abruption: an umbrella review
Source: Syst Rev. 2022 Apr 1;11:55. doi: 10.1186/s13643-022-01915-6 (PMC8973534; doi:10.1186/s13643-022-01915-6)
Supplement: Supplementary file 1 — Additional file 1: S1. Search strategy. S 2. Excluded references with reasons. S 3. Quality of studies based on AMSTAR2 items. [file 13643_2022_1915_MOESM1_ESM.docx]

**S1: Search strategy**

1. Smoking or cigarette smoking or cigarette or tobacco or cigar
2. Previous abortion or prior abortion or spontaneous abortion, induced abortion, elective abortion
3. Previous cesarean section or C(a)esarean delivery or C(a)esarean section
4. pregnancy-induced hypertension, pre-eclampsia or eclampsia or chronic hypertension
5. (myoma or myomas or leiomyoma or leiomyomas or fibroid or fibroids or fibromyoma or fibromyomas) and (uterine or uteri or uterus)
6. Parity or multiparity
7. (Mullerian or uterus or uterine) and (congenital anomalies or Mullerian anomalies or congenital abnormalities or congenital malformations)
8. Assisted reproductive technology or ART or Assisted conception or Assisted reproduction or In vitro fertilization or IVF or Test tube baby or Intracytoplasmic sperm injection or ICSI or Artificial insemination or Intrauterine insemination or IUI or Cervical canal insemination or Embryo transfer
9. Endometriosis
10. Alcohol
11. Cocaine or Marijuana
12. #1 or #2 or #3 or #4 #5 or #6 or #7 or #8 or #9 or #10 or #11
13. Placenta abruption or Placental abruption or abruptio placenta or abruptio placentae
14. Systematic review or Meta-analysis or Meta analysis or synthesis
15. #11 and #12 and #13

**S 2: Excluded references with reasons**

| ***Outcome already explored in larger meta-analysis (n=9)*** |
| --- |
| 1. Ananth CV, Smulian JC, Vintzileos AM. Incidence of placental abruption in relation to cigarette smoking and hypertensive disorders during pregnancy: A meta-analysis of observational studies. Obstetrics and Gynecology. 1999;93(4):622-8.  2. Castles A, Adams EK, Melvin CL, Kelsch C, Boulton ML. Effects of smoking during pregnancy - Five meta-analyses. American Journal of Preventive Medicine. 1999;16(3):208-15.  3. Conforti A, Picarelli S, Carbone L, La Marca A, Venturella R, Vaiarelli A, et al. Perinatal and obstetric outcomes in singleton pregnancies following fresh versus cryopreserved blastocyst transfer: a meta-analysis. Reproductive Biomedicine Online. 2021;42(2):401-12.  4. dos Santos JF, Cavalcante CDB, Barbosa FT, Gitai DLG, Duzzioni M, Tilelli CQ, et al. Maternal, fetal and neonatal consequences associated with the use of crack cocaine during the gestational period: a systematic review and meta-analysis. Archives of Gynecology and Obstetrics. 2018;298(3):487-503.  5. Gasparri ML, Nirgianakis K, Taghavi K, Papadia A, Mueller MD. Placenta previa and placental abruption after assisted reproductive technology in patients with endometriosis: a systematic review and meta-analysis. Archives of Gynecology and Obstetrics. 2018;298(1):27-34.  6. Hulse GK, Milne E, English DR, Holman CD. Assessing the relationship between maternal cocaine use and abruptio placentae. Addiction. 1997;92(11):1547-51.  7. Kobayashi H, Kawahara N, Ogawa K, Yoshimoto C. A Relationship Between Endometriosis and Obstetric Complications. Reproductive Sciences. 2020;27(3):771-8.  8. Qin J, Liu X, Sheng X, Wang H, Gao S. Assisted reproductive technology and the risk of pregnancy-related complications and adverse pregnancy outcomes in singleton pregnancies: a meta-analysis of cohort studies. Fertil Steril. 2016;105(1):73-85.e1-6.  9. Su YZ, Zhang Y, Xu ZY, Xu XF, Xing Q, Zhang LX, et al. Differences between fresh embryo transfer and frozen embryo transfer in Asian populations: a meta-analysis. International Journal of Clinical and Experimental Medicine. 2018;11(9):8820-30. |
| ***No meta-analysis (n=1)*** |
| 1. Downes KL, Grantz KL, Shenassa ED. Maternal, Labor, Delivery, and Perinatal Outcomes Associated with Placental Abruption: A Systematic Review. American Journal of Perinatology. 2017;34(10):935-57. |

**S 3: Quality of studies based on AMSTAR2 items**

| Rating | 16 | 15 | 14 | 13 | 12 | 11 | 10 | 9 | 8 | 7 | 6 | 5 | 4 | 3 | 2 | 1 | Items  Study |
| --- | --- | --- | --- | --- | --- | --- | --- | --- | --- | --- | --- | --- | --- | --- | --- | --- | --- |
| Criticaly Low | Y | Y | Y | N | N | N | N | pY | Y | N | Y | Y | pY | Y | pY | Y | Wang G |
| Criticaly Low | Y | Y | Y | N | N | N | N | pY | Y | N | Y | Y | pY | Y | Y | Y | Adane A |
| Criticaly Low | Y | Y | Y | Y | Y | N | N | pY | Y | N | N | N | pY | Y | pY | N | Klar M |
| Low | N | Y | Y | Y | N | N | N | Y | pY | Y | Y | Y | Y | Y | pY | Y | Addis A |
| Criticaly Low | Y | Y | Y | N | N | N | N | pY | Y | N | Y | Y | Y | Y | Y | N | Breintoft K |
| Criticaly Low | Y | Y | Y | N | N | N | N | pY | pY | N | Y | Y | pY | Y | pY | Y | Broere-Brown ZA |
| Criticaly Low | N | Y | Y | Y | N | N | N | pY | N | N | Y | Y | pY | Y | pY | Y | Ananth CV |
| Criticaly Low | Y | Y | Y | N | N | N | N | PY | N | N | Y | Y | Y | Y | Y | N | Jenabi E |
| Criticaly Low | N | Y | Y | Y | Y | N | N | pY | pY | N | Y | Y | pY | Y | pY | Y | Conner SN |
| Criticaly Low | Y | Y | Y | N | N | N | N | PY | pY | N | Y | Y | Y | Y | pY | Y | Martinelli KG |
| Criticaly Low | Y | Y | Y | N | N | Y | N | Y | N | N | Y | Y | Y | Y | Y | N | Shobeiri F |
| Criticaly Low | Y | Y | Y | Y | N | N | Y | pY | Y | N | Y | Y | pY | Y | Y | Y | Vermey BG |

Y: Yes; pY: Partial yes; N: No

1- PICO considered in the research question and inclusion criteria? 2- Protocol was established beforehand? Any deviations? 3- Explained if/why only certain study designs were included? 4- Comprehensive search? 5- Two persons performed the search? 6- Two persons extracted the data? Provided the exclusion list with reasons? 7- All details of the included papers presented? 8- Proper technique for assessing the risk of bias? 9- Reported the sources of funding? 10- Appropriate statistical methods? 11- Assessment of the potential impact of risk of bias in individual studies on the results of the meta-analysis? 12- Assessment of the potential impact of risk of bias in individual studies on the discussion of the meta-analysis? 13- Discussion of heterogeneity of the results of meta-analysis? 14- Investigation of publication bias? 15- Have they influenced the results? 16- Potential conflict of interest reported?
